# Climate change and the emergence of fungal pathogens

**DOI:** 10.1371/journal.ppat.1009503

**Published:** 2021-04-29

**Authors:** Nnaemeka Emmanuel Nnadi, Dee A. Carter

**Affiliations:** 1 Department of Microbiology, Faculty of Natural and Applied Scieneces, Plateau State University, Bokkos, Nigeria; 2 School of Life and Environmental Sciences, ARC Training Centre for Food Safety in the Fresh Produce Industry and the Marie Bashir Institute for Infectious Diseases and Biosecurity, The University of Sydney, Sydney, Australia; University of Maryland, Baltimore, UNITED STATES

The role of the environment in emerging and reemerging infectious diseases is increasingly recognized [1,2]. Climate change, defined by the United Nations Framework Convention on Climate Change as “a change of climate which is attributed directly or indirectly to human activity that alters the composition of the global atmosphere and which is in addition to natural climate variability observed over comparable time periods” [3] may create environmental pressures that result in new diseases caused by fungi [4]. While viral and bacterial diseases receive most attention as the potential cause of plagues and pandemics, fungi can arguably pose equal or even greater threats: There are no vaccines available yet for fungal pathogens, the arsenal of antifungal agents is extremely limited, and fungi can live saprotrophically, producing large quantities of infectious spores and do not require host-to-host contact to establish infection [5]. Indeed, fungi seem to be uniquely capable of causing complete host extinction [6]. For the vast majority of fungal species, the capacity to grow at elevated temperatures limits their ability to infect and establish in mammals. However, fungi can be trained to evolve thermotolerance, and gradual adaptation to increasing temperature caused by climate change could lead to an increase of organisms that can cause disease [7,8]. In addition, climate change can increase the geographic range of pathogenic species or their vectors, leading to the emergence of diseases in areas where they have not previously been reported [7]. Environmental disruptions due to climate change such as floods, storms, and hurricanes can disperse and aerosolize fungi or implant them via traumatic wounds, resulting in infections by previously very rare or unknown fungal species. Fig 1 summarizes the potential effects of climate change, showing examples of emerging fungi and their consequences, along with the potential for new and currently unknown species to emerge.

**Fig 1:**
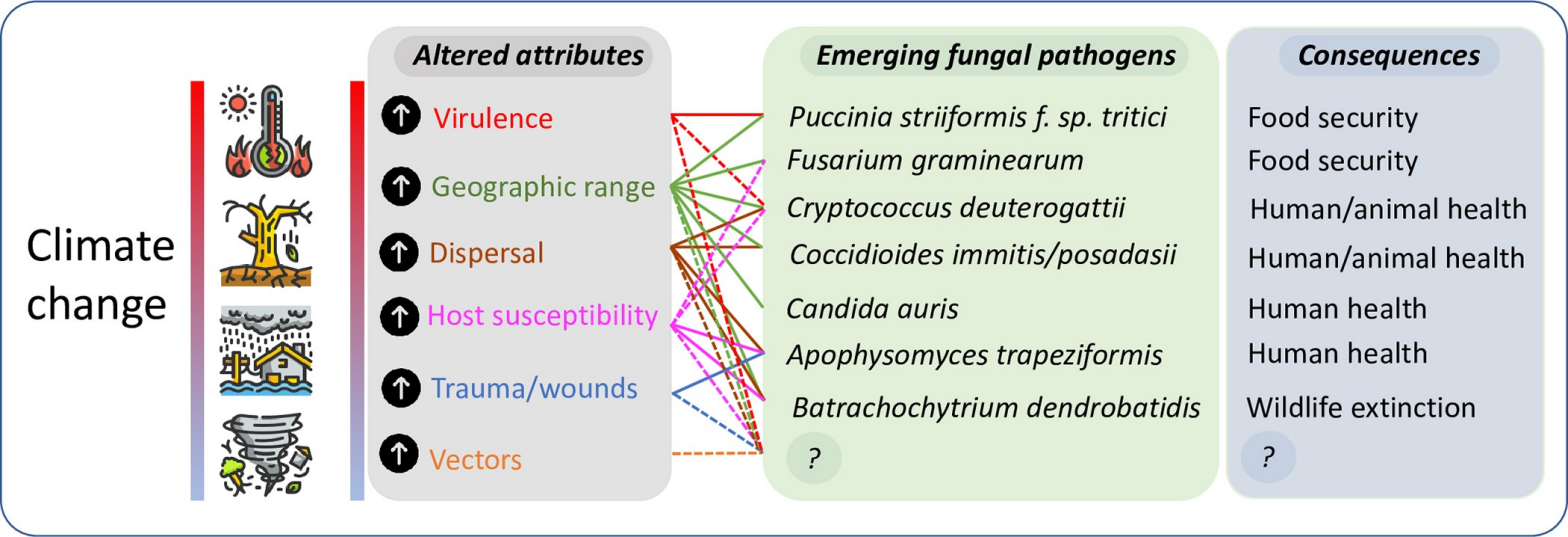
The effect of climate change on the emergence of fungal pathogens. Climate change alters attributes of the fungus, the environment, and the host, which can then drive the emergence of novel, uncommon, or adapted fungal species, with consequences for health, biodiversity, and food security. On this figure, solid lines between attributes and fungal species show links supported by published evidence; dashed lines show probable but unproven links. “?” represents the emergence of as-yet unknown fungal species and unknown consequences.

Here, we review current literature on the role of climate change in the emergence of fungal diseases. In the context of this review, we consider emerging fungal pathogens to be those that have been reported to have caused disease in plants or animals in the last 30 years, have arisen in novel geographical locations, or have become substantially more problematic, with demonstrated evidence that this emergence is attributed to climate change.

## Completely new fungal species hypothesised to have emerged due to climate change

### Candida auris

First identified in 2009 from an ear infection in Japan [9], *C*. *auris* has since spread globally, with reports from every continent [10]. Little is known about the origins of *C*. *auris*; however, its phylogentic relatives have been isolated from the environment [11], and it is hypothesized to have a nonhuman environmental reservoir with possible dispersal by birds [12]. This yeast is considered the first “novel” pathogen to have evolved in response to climate change [10,12], although this remains speculative and awaits conclusive evidence; alternative hypotheses for its emergence include expanded farming and aquaculture resulting in increased contact with humans, and environmental contamination with fungicides [11]. *C*. *auris* is particularly problematic in healthcare settings where it colonises and spreads to cause nosocomial outbreaks, and it is remarably resistant to antifungals and disinfectants [13]. Fortunately, *C*. *auris* is relatively low in virulence and has only caused infection in people suffering from severe comorbidities [12,13]. Four major genomic clades have been identified for *C*. *auris* and these are associated with particular geographic regions: clade I (South Asian), clade II (East Asian), clade III (South African), and clade IV (South American) [10]. Clade II appears to infect the ear only, whereas the other clades have caused invasive infections [14].

## Fungal species that have emerged to cause significant outbreaks that appear related to climate change

### Batrachochytrium dendrobatidis

*Batrachochytrium dendrobatidis* (*Bd*) is an emerging pathogen of amphibians. An environmental chitrid fungus, *Bd* is responsible for the most spectacular loss of amphibian diversity in history [15] and since 1998 has spread to all continents where amphibians are found, decimating populations in Australia and North and South America [16]. *Bd* has also spread to Africa, with the first report in North Africa in 2011 [17]. IPCC data forecasts an increased spread of *Bd* across the northern hemisphere, particularly in the higher-latitude regions that are experiencing warming temperatures [18]. In addition to the increased global range of the pathogen, a shift from cold to hotter temperature has been shown to increase the susceptibility of the amphibian host [19]. Increasing climate change is therefore predicted to cause an ongoing decline in amphibian populations.

### Cryptococcus deuterogattii

*Cryptococcus deuterogattii*, previously named *Cryptococcus gattii* VGII, was recently elevated to a species within the *Cryptococcus gattii* species complex [20]. *C*. *gattii* complex species were traditionally associated with tropical and subtropical climates [21]; however, in recent years, *C*. *deuterogattii* has emerged in the temperate regions of western Canada and the Pacific northwest of North America where it caused hundreds of infections in people and animals [22]. Interestingly, *C*. *deuterogattii* has a higher level of thermal tolerance than other *C*. *gattii* complex members, indicating that it may have a greater capacity for thermal adaptation [23]. The spread of this fungus has been attributed to anthropogenic dispersal through vehicle wheel wells, footwear, construction and forestry activity (leading to aerial dispersal), and water [24], with climate change suggested as a potential driver [25].

## New virulent fungal lineages with adaptations suggested to have evolved in response to climate change

### Puccinia striiformis (Rust fungus)

Stripe rust is one of the most devastating global diseases of wheat and is caused by the fungus *Puccinia striiformis* [26,27]. Wheat yellow (stripe) rust, caused by *Puccinia striiformis f*. sp. *tritici* (*Pst*), has previously shown preference for cold areas but has recently been seen to invade warmer regions [28]. Since 2000, *Pst* has been reported to have adapted to increased temperatures, with novel strains Pst1, Pst2, and “Warrier,” which are more aggressive and thermotolerant, replacing older strains and spreading to new regions [28]. These strains have now been reported worldwide [30], with major outbreaks occuring in south central United States [29,31] and Australia [32]. Microsatellite genotyping and virulence phenotyping have detected a high degree of variability within the Pst1 and Pst2 lineages, demonstrating an ongoing evolutionary potential of this pathogen [30].

### *Fusarium* head blight

*Fusarium* head blight (FHB) is a disease of great concern for wheat and for other cereal crops [33]. Caused by members of the *Fusarium graminearum* species complex (FGSC) [34], infection leads to reduced cereal yield and quality, thereby negatively affecting food security. Outbreaks occur particularly in years with warm and humid weather conditions, resulting in yield losses of up to 75% [35]. Over the past approximately 20 years, some temperate regions have seen a shift from *Fusarium culmorum*, which is associated with cooler and wetter conditions, to *F*. *graminearum*, which favours warmer, humid conditions [36]. Importantly, *F*. *graminearum* is more aggressive than *F*. *culmorum* and is associated with higher yield loss [37]; hence, the severity of FHB is likely to further increase in future warmer climates. *F*. *graminearum* also produces more mycotoxins, and there is evidence that their production increases with temperature and water stress [38], with potential impacts on human and animal health.

## Fungal pathogens that have become more problematic due to environmental disruption caused by climate change

Climate change affects rainfall causing floods and drought and can increase tropical cyclone and tornado severity, leading to far-reaching human health impacts. The link between natural disasters and subsequent fungal infections in disaster-affected persons is increasingly recognized, with an excellent review of this area by Benedict and Park [39]. Disaster-associated fungi can be violently displaced and widely disseminated, leading to pulmonary and soft tissue infections by fungal species that may otherwise be uncommon. For example, a severe duststorm in the southern San Joaquin Valley of California in 1977 dispersed *Coccidioides immitis* from Bakersfield, an area of high endemnicity, to Sacremento County, where it is rare, resulting in more than 100 infections [40]. While this predates the era of significant climate change, it demonstrates the ramifications of acute environmental disruption, particularly as climate change modelling predicts that the range of *Coccidioides* could expand from 12 to 17 states, with infections increasing by 50% [41] *Apophysomyces trapeziformis*, a thermotolerant saprotroph that is an extremely rare cause of mucormycosis caused a cluster of cases in patients wounded following the 2011 tornado in Joplin, Missouri [42]. Various other soil-borne fungal pathogens have been speculated to have increased in frequency or range due to climate-induced disruptions, including *Talaromyces marneffei* [43], *Blastomyces*, *Histoplasma* [44], and *Paracoccidioides* [45]. Outside of their normal range, these fungal infections can be challenging to diagnose and they are often refractory to treatment, frequently resulting in poor patient outcomes [40].

## Conclusions and future perspectives

Unless action is taken to drastically reduce carbon emissions, the global temperature will continue to rise, and the impacts of climate change are predicted to become increasingly severe. Fungi are likely to continue to affect crops and native plants, with expanding ecological range and long-distance dispersal events producing new risks. The threat of novel fungal infections of humans and other mammals is less predictable, and there is a need to understand more about the nexus between ecology and medical mycology, which have traditionally been studied in relative isolation [46]. It is now recognised that many clinically relevant fungal pathogens, including those traditionally considered to be human commensals like *Candida albicans*, can be found in the environment [47], with environmental pressures affecting the evolution of novel traits including virulence and antifungal resistance [48].

Climate change produces a confluence of factors that can act together to drive the emergence of new pathogens (Fig 1). In the clinical setting, it may become increasingly necessary to consider infection by novel or uncommon species, or adapted strains within species, which might otherwise be considered unlikely to be endemic or capable of breaching the mammalian thermal barrier. The era of HIV-AIDS and immunosuppression rewrote the textbooks on medical mycology and what may or may not be considered a potential fungal pathogen; it is likely that climate change will do this again.
